# Expanding the phenotype of *CACNA1C* mutation disorders

**DOI:** 10.1002/mgg3.1673

**Published:** 2021-04-01

**Authors:** Lindsey Gakenheimer‐Smith, Lindsay Meyers, Derek Lundahl, Shaji C. Menon, T. Jared Bunch, Briana L. Sawyer, Martin Tristani‐Firouzi, Susan P. Etheridge

**Affiliations:** ^1^ Division of Pediatric Cardiology University of Utah Salt Lake City Utah USA; ^2^ Genome Medical San Francisco California USA; ^3^ Division of Cardiovascular Medicine University of Utah University of Utah Health Sciences Center Salt Lake City Utah USA

**Keywords:** atrial fibrillation, *CACNA1C*, cardiac‐only Timothy syndrome, sick sinus syndrome

## Abstract

**Background:**

Pathogenic variants in the L‐type Ca^2+^ channel gene *CACNA1C* cause a multi‐system disorder that includes severe long QT syndrome (LQTS), congenital heart disease, dysmorphic facial features, syndactyly, abnormal immune function, and neuropsychiatric disorders, collectively known as Timothy syndrome. In 2015, a variant in *CACNA1C* (p.R518C) was reported to cause cardiac‐only Timothy syndrome, a genetic disorder with a mixed phenotype of congenital heart disease, hypertrophic cardiomyopathy (HCM), and LQTS that lacked extra‐cardiac features. We have identified a family harboring the p.R518C pathogenic variant with a wider spectrum of clinical manifestations.

**Methods:**

A four‐generation family harboring the p.R518C pathogenic variant was reviewed in detail. The proband and his paternal great‐uncle underwent comprehensive cardiac gene panel testing, and his remaining family members underwent cascade testing for the p.R518C pathogenic variant.

**Results:**

In addition to displaying cardinal features of *CACNA1C* disorders including LQTS, congenital heart disease, HCM, and sudden cardiac death, family members manifested atrial fibrillation and sick sinus syndrome.

**Conclusion:**

Our report expands the cardiac phenotype of *CACNA1C* variants and reflects the variable expressivity of mutations in the L‐type Ca^2+^ channel.

## Graphical abstract

We present a four‐generation family harboring the *CACNA1C* p.R518C pathogenic variant that, in addition to displaying cardinal features of *CACNA1C* disorders including Long QT syndrome, congenital heart disease, hypertrophic cardiomyopathy, and sudden cardiac death, also manifest atrial fibrillation and sick sinus syndrome. Our report expands the cardiac phenotype of *CACNA1C* variants and reflects the variable expressivity of mutations in the L‐type calcium channel.

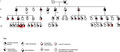

## 1 INTRODUCTION

Pathogenic variants in the L‐type Ca^2+^ channel gene *CACNA1C* (OMIM 114205) were first identified in a series of patients manifesting a multi‐system disorder that included severe long QT syndrome (LQTS), congenital heart disease, dysmorphic facial features, syndactyly, abnormal immune function, and neuropsychiatric disorders, collectively known as Timothy syndrome (OMIM 601005) (Splawski et al., 1993). In 2015, several families were ascertained harboring *CACNA1C* variants with unique biophysical features, c.1552C>T (p.Arg518Cys) and c.1553G>A (p.Arg518His), referred to as p.R518C and p.R418H, respectively, through the remainder of the paper. These two variants have been reported to result in cardiac‐specific phenotypes, the so‐called cardiac‐only Timothy syndrome (Boczek et al., 2015). Members of these families manifested LQTS, congenital heart disease, hypertrophic cardiomyopathy (HCM), and sudden cardiac death, but not dysmorphic or neuropsychiatric disorders.

Here, we expand the cardiac phenotypes of *CACNA1C* pathogenic variants with the description of a four‐generation family harboring the p.R518C *CACNA1C* variant that segregated with phenotypes in a highly penetrant autosomal dominant manner. While this family carried diagnoses of HCM, LQTS, congenital heart disease, and sudden cardiac death, some individuals also developed atrial fibrillation (AF) and sick sinus syndrome. Family members did not have other extra‐cardiac manifestations characteristic of Timothy syndrome (Splawski et al., 1993). Our report expands the cardiac phenotype of *CACNA1C* variants and underscores the variable expressivity of *CACNA1C* disorders.

### 1.1 Ethical compliance

This study was approved by the University of Utah Ethics Committee.

### 1.2 Index case

The index case (IV.5) is a male who presented at 59 days of age with a murmur. An electrocardiogram showed left axis deviation and a prolonged QTc at 472 ms. An echocardiogram showed features consistent with HCM with significant septal hypertrophy (M‐mode intraventricular septum measurement in diastole 1.23 cm (Z‐score 11.23)) and severe dynamic left ventricular outflow tract obstruction (gradient 72 mmHg). There was evidence of systolic anterior motion of the mitral valve. He also had a small ventricular septal defect. He was started on beta‐blocker therapy at that time and has remained on beta‐blockers. He underwent implantable cardioverter‐defibrillator (ICD) placement at four months of age due to multiple syncopal episodes presumed to be arrhythmic. At three years of age, he underwent extended left ventricular septal myectomy, resection of subaortic membrane, and ventricular septal defect closure. Five years later, he has exercise intolerance, mild outflow tract obstruction, and mild mitral valve regurgitation. He has not had further syncopal episodes, ICD shocks, or evidence of arrhythmias. His QTc remains prolonged at 493 ms.

### 1.3 Pedigree

**FIGURE 1:**
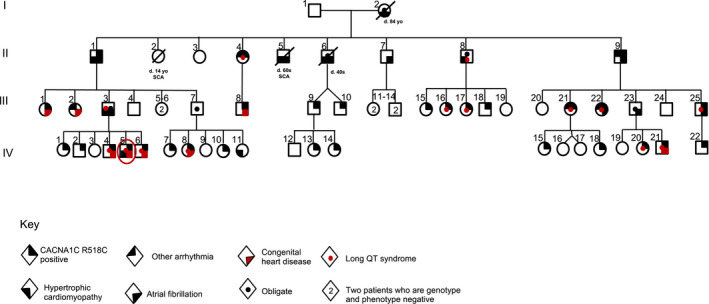
Single‐family pedigree of patients harboring the *CACNA1C* p.R518C pathogenic variant and their associated diagnoses (key below figure). The red circle indicates the index patient

A review of a four‐generation pedigree (Figure 1) is notable for multiple family members with a variety of structural and conduction cardiac defects (Table 1). The index patient's father (III.3) had HCM diagnosed on a screening cardiac MRI at 28 years of age and AF identified at 30 years of age. He also has LQTS. A paternal grandfather (II.1) was diagnosed with lone AF around 20 years of age and subsequently diagnosed with HCM at 51 years of age. The proband's father and paternal grandfather underwent ICD implantation for primary prevention of sudden cardiac death. A paternal great aunt (II.2) experienced a cardiac arrest as an infant and died suddenly at 14 years of age from a presumed cardiac arrest. A paternal aunt (III.2) was born with subaortic stenosis requiring surgical resection and a modified Konno procedure. She developed a surgical complete heart block requiring dual‐chamber pacemaker implantation. A few years later, she was found to have pre‐excitation on routine electrocardiograms suspicious for Wolff‐Parkinson‐White syndrome (WPW). She underwent an electrophysiology study that confirmed the presence of an accessory atrioventricular conduction pathway with anterograde‐only conduction. An ablation was not required because the pathway was incapable of rapid anterograde conduction and there was no evidence of retrograde conduction across an accessory pathway or AV nodal conduction during the electrophysiology study. At 20 years of age, her pacemaker was upgraded to an ICD after the identification of non‐sustained ventricular tachycardia during a device interrogation. Five years later, she was found to have heart failure with a preserved ejection fraction.

**TABLE 1:** Characteristics and diagnoses of genotype‐positive, phenotype‐positive, or obligate carriers of the *CACNA1C* p.R518C pathogenic variant

Patient	Gene testing (Y/N)	Age at diagnosis (years)	Age at death (years)	Max QTc (ms)	Heart rate (bpm)	Congenital heart disease	Sick sinus syndrome	ICD (Y/N)	Other diagnoses
I.2	N (obligate carrier)	Unknown	84	—	—	—	—	Y	AF, HCM
II.1	Y	~20	—	468	60	—	—	Y	AF, HCM
II.2	N	0.5	14	—	—	—	—	N	SCD
II.4	Y	47	—	491	53	—	Brady‐arrhythmia	N (declined)	Systolic anterior motion of mitral valve without HCM
II.5	N	49	55	—	—	—	—	N	AF, HCM, SCD
II.6	N (obligate carrier)	Unknown	44	—	—	—	—	Unknown	HCM, AF
II.7	N	52	—	446	60	—	—	Y	AF, borderline LVH (1.3 cm)
II.8	N (obligate carrier)	26	—	480	69	—	—	Y	HCM
II.9	Y	20	—	N/A (paced)	—	—	Tachy Brady syndrome	Y	AF
III.1	Y	4	—	458	58	VSD	—	N	—
III.2	N	Within 60 days of life	—	N/A (paced)	—	Subaortic stenosis, VSD	—	Y	Surgical Heart Block, WPW
III.3	N (obligate carrier)	28	—	475	72	—	—	Y	AF, HCM
III.7	N (obligate carrier)	N/A	—	447	70	—	—	—	—
III.8	Y	10	—	452	57	PDA	—	N	—
III.9	Y	N/A	—	444	83	—	—	—	—
III.10	Y	N/A	—	—	—	—	—	—	MRI findings of heterogeneous late gadolinium enhancement in ventricles
III.15	Y	N/A	—	457	52	—	—	—	—
III.16	Y	1.5	—	475	72	—	—	N	Systolic anterior motion of mitral valve without LVH
III.17	Y	18	—	486	66	—	—	N	—
III.18	Y	N/A	—	420	134	—	—	N	—
III.21	Y	35	—	528	66	—		Y	Atrial Tachycardia
III.22	Y	24	—	470	41	—	Tachy Brady Syndrome	Y	AF
III.23	N (obligate carrier)	29	—	452	80	—	—	N	AF
III.25	Y	27	—	507	63	—	—	Y	AF, mild LVH (1.3 cm)
IV.1	Y	N/A	—	467	82	—	—	—	—
IV.2	Y	N/A	—	467	67	—	—	—	—
IV.4	Y	39 days	—	483	79	VSD	—	N	—
IV.5	Y	49 days	—	536	104	VSD	—	Y	HCM
IV.6	Y	90 days	—	478	136	VSD	—	N	—
IV.7	Y	N/A	—	464	60	—	—	—	—
IV.8	Y	11 days	—	481	72	ASD	—	N	—
IV.10	Y	N/A	—	445	63	—	—	—	—
IV.11	N	1.5	—	N/A (LBBB)	—	—	—	N	HCM
IV.13	Y	N/A	—	—	—	—	—	—	—
IV.14	Y	N/A	—	—	—	—	—	—	—
IV.15	Y	N/A	—	447	80	—	—	—	—
IV.18	Y	N/A	—	463	57	—	—	—	—
IV.20	Y	2	—	483	82	—	—	N	—
IV.21	Y	Prenatal	—	485	117	HLHS	—	N	Perioperative SVT
IV.22	Y	N/A	—	459	107	—	—	—	—

Patients IV.5 (the proband) and II.9 underwent genetic testing with a full genetic panel. For the remaining patients who underwent genetic testing, cascade testing was performed. All patients who underwent genetic testing were positive for the *CACNA1C* p.R518C pathogenic variant.

Abbreviations: AF, atrial fibrillation; ASD, atrial septal defect; HCM, hypertrophic cardiomyopathy; HLHS, hypoplastic left heart syndrome; MRI, magnetic resonance imaging; PDA, patent ductus arteriosus; SCD, sudden cardiac death; SVT, supraventricular tachycardia; VSD, ventricular septal defect; WPW, Wolff‐Parkinson‐White syndrome.

Table 1 lists the phenotype of affected patients. In total, two family members have died from sudden cardiac death (II.2, II.5), ten have AF refractory to ablation (I.2, II.1, II.5–7, II.9, III.3, III.22–23, III.25), eight have HCM (I.2, II.1, II.5–6, II.8, III.3, IV.5, IV.11), and 14 relatives have LQTS (II.4, II.8, III.3, III.16, III.17, III.21, III.22, III.25, IV.4, IV.5, IV.6, IV.8, IV.20, IV.21). Three relatives have sick sinus syndrome manifested as bradyarrhythmia (II.4) or tachy‐brady syndrome (II.9, III.22). Eight relatives have congenital heart disease including hypoplastic left heart syndrome (IV.21), subaortic stenosis (III.2), ventricular or atrial septal defects (III.1, III.2, IV.4‐6, IV.8), and patent ductus arteriosus (III.8).

Each patient who received genetic testing was evaluated by a genetic counselor. One patient has autism, but the other genotype‐positive patients have not displayed non‐cardiac manifestations described in *CACNA1C* pathogenic variants including seizures, musculoskeletal abnormalities, or immunodeficiencies. Many genotype‐positive family members have anxiety and depression, but we have not screened genotype‐negative family members for these mental health conditions to be able to definitively conclude that mental health conditions segregate with the *CACNA1C* pathogenic variant in this family. As only one family member in this large pedigree has autism, we feel we cannot definitively conclude that the p.R518C pathogenic variant is responsible for this patient's autism.

### 1.4 Genetic testing

Human subjects research for this study, including genetic testing and review of medical records, was approved by the University of Utah Institutional Review Board (IRB_00021080). Genetic testing on the index patient was performed under routine clinical care, using a commercial comprehensive cardiac gene panel (Invitae Laboratories). Genomic DNA was collected from whole blood samples on our index patient and his at‐risk family members. The proband and his paternal great‐uncle (II.9) underwent comprehensive cardiac gene panel testing with a cardiomyopathy and arrhythmia panel (Table 2). Identification of the *CACNA1C* p.R518C variant in the proband and his paternal great‐uncle and cascade testing of the at‐risk family members was performed through Invitae laboratories, a CLIA certified diagnostic lab.

**TABLE 2:** Two patients with a different cardiac phenotype underwent a cardiomyopathy (II.9 and IV.5) and arrhythmia (II.9) genetic panel identifying the same *CACNA1C* p.R518C pathogenic variant

Patient	Phenotype	Genes tested	Variant(s) Identified and ACMG Classification (Richards et al., 2015)
II.9	Atrial Fibrillation, Tachy Brady Syndrome	ABCC9, ACTC1, ACTN2, AKAP9, ALMS1, ANK2, ANKRD1, BAG3, CACNA1C, CACNA2D1, CACNB2, CALM1, CALM2, CALM3, CALR3, CASQ2, CAV3, CRYAB, CSRP3, CTF1, CTNNA3, DES, DMD, DSC2, DSG2, DSP, DTNA, ELAC2, EMD, EYA4, FHL1, FHL2, FKRP, FKTN, GATA4, GATA6, GATAD1, GLA, GPD1L, HCN4, ILK, JPH2, JUP, KCND3, KCNE1, KCNE2, KCNE3, KCNE5, KCNH2, KCNJ2, KCNJ5, KCNJ8, KCNQ1, LAMA4, LAMP2, LDB3, LMNA, MTO1, MYBPC3, MYH6, MYH7, MYL2, MYL3, MYLK2, MYOM1, MYOZ2, MYPN, NEBL, NEXN, NKX2‐5, NPPA, PDLIM3, PKP2, PLN, PRDM16, PRKAG2, RAF1, RANGRF, RBM20, RYR2, SCN10A, SCN1B, SCN2B, SCN3B, SCN4B, SCN5A, SGCD, SLMAP, SNTA1, TAZ, TCAP, TGFB3, TMEM43, TMPO, TNNC1, TNNI3, TNNT2, TPM1, TRDN, TRPM4, TTN, TTR, TXNRD2, VCL	CACNA1C c.1552C>T (p.Arg518Cys) – pathogenic TRPM4 c.2891G>A (p.Arg964His) – uncertain significance
IV.5	Hypertrophic Cardiomyopathy, Ventricular Septal Defect, Long QT Syndrome	ACTC1, ACTN2, AGL, ANKRD1, BAG3, CALR3, CAV3, CSRP3, DES, FHL1, FLNC, GAA, GATA4, GLA, JPH2, LAMP2, LDB3, MYBPC3, MYH6, MYH7, MYL2, MYL3, MYLK2, MYOM1, MYOZ2, MYPN, NEXN, PDLIM3, PLIN, PRKAG2, TCAP, TNNC1, TNNI3, TNNT2, TPM1, TTR, VCL	CACNA1C c.1552C>T (p.Arg518Cys) – pathogenic

The heterozygous pathogenic variant, c.1552C>T (p.Arg518Cys), in exon 12 of the *CACNA1C* gene, was present in our index patient, his paternal great uncle, and 27 of their relatives. The p.R518C variant substitutes a positively charged arginine residue for a polar, neutral cysteine and was found to alter the properties of the Ca_V_1.2 L‐type Ca^2+^ channel (Boczek et al., 2015). This region is conserved during evolution, and the c.1552C>T variant is not present in the Exome Aggregation Consortium (Lek et al., 2016) or the NHLBI Exome Sequencing Project (http://evs.gs.washington.edu/EVS/). This variant has also been identified in three unrelated families with cardiac‐only Timothy syndrome and segregated with disease (Boczek et al., 2015). The p.R518C variant is considered pathogenic, based on ACMG classification (Richards et al., 2015).

At the time of this writing, the penetrance of the p.R518C pathogenic variant in this family is at least 72%, with 21 of the 29 gene positive family members or obligate carriers displaying a positive phenotype. Three genotype‐positive patients were lost to follow‐up, so their phenotype is unknown. Four phenotype positive patients have not undergone genetic testing due to patient preference or death prior to genetic testing availability.

## 2 DISCUSSION

Timothy syndrome is unique among channelopathies in that the associated phenotypes are not only complex but have varied widely since the original description (Napolitano et al., 2006). Although originally described as severe LQTS and syndactyly, the ascertainment of additional subjects revealed a more complex clinical constellation, including congenital heart disease, neuropsychiatric disorders (including autism), abnormal dentition, and facial dysmorphic features. All members of the original Timothy syndrome cohort displayed severe QT prolongation, syndactyly, baldness at birth, small teeth, and immunodeficiency, and, remarkably, carried the identical, *de novo* missense variant in the alternatively spliced exon 8A (G406R) (Splawski et al., 2004). Shortly thereafter, Timothy syndrome cases notable for the absence of the cardinal feature syndactyly were reported in patients harboring variants in the dominant splice variant exon 8, p.G402S and p.G406R, called Timothy syndrome type 2. These patients displayed severe symptoms including seizures, arrhythmias, and intellectual disability (Splawski et al., 2005). Since then, pathogenic variants in various regions of the L‐type Ca^2+^ channel have been described in individuals manifesting more limited clinical phenotypes, such as “LQTS‐only” and “cardiac‐only” Timothy syndrome (Boczek et al., 2013, 2015). In this report, we further expand the cardiac phenotype of *CACNA1C* pathogenic variants to include AF and sick sinus syndrome.

The *CACNA1C* gene encodes for the α‐subunit of the Ca_V_1.2 L‐type Ca^2+^ channel. The p.R518C amino acid is located within the I‐II linker that includes the α‐interaction domain where the Ca_V_1.2 β‐subunit binds. The binding of these subunits is crucial for many Ca_V_1.2 L‐type Ca^2+^ channel functions including voltage‐dependent activation, G‐protein modulation, and cell surface expression (Van Petegem et al., 2004). In cardiac tissue, the L‐type Ca^2+^ channel plays a key role in the plateau phase of the cardiac action potential, excitation‐contraction coupling, cardiac β‐adrenergic regulation, and regulation of gene expression (Benitah et al., 2010; Lu et al., 2015). *CACNA1C* pathogenic variants cause a range of cardiac diseases that can be dichotomized by gain‐of‐function versus loss‐of‐function mutations.

Interestingly, the *CACNA1C* p.R518C mutant channel exhibits both loss‐of‐function and gain‐of‐function properties (Boczek et al., 2015). L‐type Ca^2+^ channels expressed by this altered gene exhibit a reduction in current density secondary to a trafficking defect that impedes cell surface expression. The gain‐of‐function properties include a reduction in the rate of current inactivation and a shift in the inactivation curves to more depolarized potentials (Boczek et al., 2015). Using a human pluripotent stem cell model of the *CACNA1C* p.R518C variant, it was confirmed that these gain‐of‐function properties cause delayed repolarization and prolonged action potential duration and, thus, the LQTS phenotype (Estes et al., 2019). It is speculated that the unique biophysical characteristics of the p.R518C variant, that is, the combination of loss‐ and gain‐of‐function, might explain the cardiac‐only phenotypes observed in these individuals (Boczek et al., 2015). Additionally, alternative splicing might also contribute to the variable phenotypes described in these patients.

In addition to displaying cardinal features of *CACNA1C* disorders, including LQTS, sudden cardiac death, congenital heart disease, and HCM, ten carriers of the p.R518C pathogenic variant in our family developed young‐onset AF refractory to ablation. Five individuals were later diagnosed with HCM, which is a known risk factor for AF (Darbar & Roden, 2013). One could speculate that these individuals may have suffered abnormal ventricular relaxation before the onset of overt HCM, which then contributed to the young‐onset AF. However, for the remaining five pathogenic variant carriers, young‐onset AF appears to be the primary diagnosis, thus implicating *CACNA1C* as an AF susceptibility gene. Clinical and experimental models of AF reveal a reduction in *CACNA1C* transcripts and protein levels (Zhao et al., 2016), a phenomenon contributing to the concept that “AF begets AF” (Wijffels et al., 1995). These observations are consistent with the finding that p.R518C mutant channels do not effectively traffick to the cell surface in heterologous expression systems (Boczek et al., 2015). Further research is required to fully elucidate the contribution of *CACNA1C* variants to the mechanisms underlying AF.

Three p.R518C *CACNA1C* carriers in our pedigree developed sick sinus syndrome manifested as sinus pauses, bradyarrhythmia, and tachy‐brady syndrome. To our knowledge, there is only one other report of sick sinus syndrome in patients with *CACNA1C* variants. Zhu et al., (2018) described a family with two *CACNA1C* variants (p.V596M and p.A1782T) and a variant in *TTN* (p.R16472H) who developed sinus bradycardia, AF, and early repolarization. Functional studies were not performed and, thus, the contribution of these variants to the reported phenotypes remains unclear (Zhu et al., 2018). There are no published studies evaluating the effect of *CACNA1C* genetic variants on sinus node function. However, the presence of Ca_V_1.2 L‐type Ca^2+^ channels in the sinoatrial node, albeit at a smaller concentration compared to Ca_V_1.3 Ca^2+^ channels, is well described (Zamponi et al., 2015). Thus, it is plausible that the *CACNA1C* p.R518C variant alters Ca_V_1.2 channel function in the sinoatrial node to create sick sinus syndrome in some mutation carriers.

It is important to note that one family member with sick sinus syndrome carries a variant in the *TRPM4* gene (p.R964H) which is expressed in sinoatrial nodal tissue (Demion et al., 2007). However, based on ACMG classification (Richards et al., 2015), it is a variant of uncertain significance. The two other patients with sick sinus syndrome were not tested for the *TRPM4* variant, so we do not know if this variant is segregated with the phenotype of sinoatrial node dysfunction. Regardless, as this variant is classified as of uncertain significance, at the time of this writing, it cannot be considered causal in this patient's sick sinus syndrome.

Variants in *TRPM4* have been linked to cardiac conduction diseases, primarily right bundle branch block and atrioventricular block (Stallmeyer et al., 2012). In addition, mice with a homozygous *TRPM4* deletion exhibited alterations in action potential duration at the sinoatrial node, suggesting that *TRPM4* contributes to heart rate modulation at the sinoatrial node (Hof et al., 2013). To date, there are no reports of *TRPM4* variants in humans with sinoatrial conduction disease. However, the aforementioned supporting evidence of the role of *TRPM4* in modulating mammalian cardiac conduction suggests that our patient's sick sinus syndrome may be related to the *TRPM4* variant instead of the *CACNA1C* variant. It is also plausible that the two variants act synergistically to disrupt normal cardiac conduction at the sinoatrial node. Because of the limited data in our pedigree and in the current scientific literature, we can only speculate the role of the *TRPM4* p.R964H and *CACNA1C* p.R518C variants in our patients’ sick sinus syndrome.

Of note, one patient in our study cohort developed WPW. This patient is uninterested in genetic testing, so we do not know if she carries the *CACNA1C* variant. However, there are two reports of WPW in two patients with *CACNA1C* variants. The first report described a patient with known WPW who died of sudden cardiac death. A whole‐exome sequencing molecular autopsy revealed rare variants in exons 43 and 45 of *CACNA1C* plus variants in six other genes associated with cardiac channelopathies (Qiu et al., 2018). The second case included the identification of the *CACNA1C* variant c.2579G>A (p.Arg860Gln) in a patient with WPW, classified as a variant of unknown significance in Clinvar (Landrum et al., 2018). Most cases of WPW have been linked to pathogenic variants in *PRKAG2* or *MYH6*, glycogen storage disorders, mitochondrial syndromes, or congenital heart diseases such as septal defects, Ebstein malformation of the tricuspid valve, or HCM (Bowles et al., 2015; Ehtisham & Watkins, 2005). We acknowledge it is speculative to presume an association between *CACNA1C* variants and WPW based on the limited data in our study. However, because this is the third potential association between WPW and *CACNA1C*, we feel it warrants mentioning to spark further discussion and research into these correlations.

Through this four‐generation pedigree, we have expanded the phenotype of *CACNA1C* disorders to include AF and sick sinus syndrome. While genotype–phenotype correlations suggest that variants in specific regions of the L‐type Ca^2+^ channel confer specific phenotypes, the genetic background of the individual may modulate channel function to cause such variable expressivity. We conclude that *CACNA1C* pathogenic variants causing LQTS‐only or cardiac‐only phenotypes reflect the variable expressivity of mutations in the L‐type Ca^2+^ channel.

## CONFLICTS OF INTEREST

The authors have no conflicts of interest or financial support to disclose.

## AUTHOR’S CONTRIBUTIONS

LGS and LM conceptualized, planned, and drafted the manuscript. SPE and MTF contributed to the design, writing, and revision of the manuscript. DL, BS, SM, and TJB participated in data collection and revision of the manuscript. All authors read and approved the final manuscript.

## Data Availability

The data that support the findings of this study are available on request from the corresponding author. The data are not publicly available due to privacy or ethical restrictions.
